# 434. Characteristics and long-term outcomes of SARS-CoV-2 associated myocarditis cases in the Military Health System

**DOI:** 10.1093/ofid/ofad500.504

**Published:** 2023-11-27

**Authors:** Simon Pollett, Stephanie A Richard, Catherine Berjohn, Josh Chenoweth, Paul Blair, Clifton Dalgard, Nusrat J Epsi, Margaret Sanchez Edwards, Allison M Malloy, Ryan Flanagan, Milissa U Jones, Anuradha Ganesan, Nikhil Huprikar, David Lindholm, Anthony C Fries, Katrin Mende, Rhonda E Colombo, Mark P Simons, Evan Ewers, Robert O’Connell, David Tribble, Brian Agan, Timothy Burgess, Mark Haigney

**Affiliations:** Infectious Disease Clinical Research Program, Department of Preventive Medicine and Biostatistics, Uniformed Services University of the Health Sciences, Bethesda, MD, USA, Bethesda, Maryland; Infectious Disease Clinical Research Program, Department of Preventive Medicine and Biostatistics, Uniformed Services University of the Health Sciences, Bethesda, MD, USA, Bethesda, Maryland; Naval Medical Center San Diego, San Diego, California; Henry M. Jackson Foundation, Bethesda, Maryland; Austere Environments Consortium for Enhanced Sepsis Outcomes, Henry M. Jackson Foundation for the Advancement of Military Medicine, Inc, Bethesda, Maryland; The American Genome Center, Uniformed Services University of the Health Sciences, Bethesda, Maryland; Uniformed Services University of the Health Sciences, Bethesda, Maryland; Infectious Disease Clinical Research Program, Department of Preventive Medicine and Biostatistics, Uniformed Services University of the Health SciencesHenry M. Jackson Foundation for the Advancement of Military Medicine, Bethesda, Maryland; Department of Pediatrics, Uniformed Services University of the Health Sciences, Bethesda, MD, USA, Bethesda, Maryland; Tripler Army Medical Center, Honolulu, Hawaii; Uniformed Services University, Bethesda, Maryland; Infectious Disease Clinical Research Program, USUHS; Henry M. Jackson Foundation for the Advancement of Military Medicine Inc, Bethesda, Maryland; Walter Reed National Military Medical Center, Bethesda, Maryland; Department of Medicine, Uniformed Services University of the Health Sciences; Brooke Army Medical Center, San Antonio, Texas; U.S. Air Force School of Aerospace Medicine, Dayton, Ohio; 1Infectious Disease Clinical Research Program, Department of Preventive Medicine and Biostatistics, Uniformed Services University of the Health Sciences and Brooke Army Medical Center, JBSA Fort Sam Houston, TX, San Antonio, TX; Infectious Disease Clinical Research Program, USUHS; Henry M. Jackson Foundation for the Advancement of Military Medicine, Inc., Bethesda, Maryland; Infectious Disease Clinical Research Program, Department of Preventive Medicine and Biostatistics, Uniformed Services University of the Health Sciences, Bethesda, MD, USA, Bethesda, Maryland; Fort Belvoir Community Hospital, Fort Belvoir, Virginia; Infectious Disease Clinical Research Program, USUHS, Bethesda, Maryland; Infectious Disease Clinical Research Program, Department of Preventive Medicine and Biostatistics, Uniformed Services University of the Health Sciences, Bethesda, MD, USA, Bethesda, Maryland; Infectious Disease Clinical Research Program, Department of Preventive Medicine and Biostatistics, Uniformed Services University of the Health Sciences, Bethesda, MD, USA, Bethesda, Maryland; Infectious Disease Clinical Research Program, Department of Preventive Medicine and Biostatistics, Uniformed Services University of the Health Sciences, Bethesda, MD, USA, Bethesda, Maryland; Military Cardiovascular Outcomes Research, Department of Medicine, Uniformed Services University of the Health Sciences, Bethesda, MD, USA, Bethesda, Maryland

## Abstract

**Background:**

SARS-CoV-2 myocarditis is a complication of COVID-19 and it is important to clarify the risk, clinical characteristics, and long-term outcomes of this complication. It is uncertain whether subsequent SARS-CoV-2 reinfections or COVID-19 vaccine administration after COVID-19-associated myocarditis can trigger a recurrence of myocarditis.

**Methods:**

We examined 5265 Military Health System (MHS) beneficiaries who tested positive for SARS-CoV-2 and enrolled into the Epidemiology, Immunology and Clinical Characteristics of Emerging Infectious Diseases with Pandemic Potential (EPICC) study between March 2020 and April 2022. Possible post-SARS-CoV-2 myocarditis cases were identified using ICD-10 codes, with further medical record adjudication and COVID-19 attribution by a cardiologist using CDC diagnostic criteria. Long-term survival and major myocarditis complications were examined in the medical record.

**Results:**

We identified 11 probable post-COVID-19 myocarditis cases, comprising 0.21% (95% CI: 0.10 to 0.37%) of all evaluated SARS-CoV-2 cases. The median age was 28.0 years (IQR = 21.5, 45.8; range = 15.0 to 63.6), and 63.6% were male. Among the myocarditis cases, elevated BMI was common (7/11, 63.6% overweight/obese), and comorbidities were otherwise rare. At the time of initial infection, 10/11 (90.9%) were unvaccinated. There were no deaths by one year post enrollment. Left ventricular dysfunction complicated 3 out of 11 cases (27%), and chronic LV dysfunction developed in one of these cases. One case was complicated by non-sustained polymorphic ventricular tachycardia without further recurrence. Following myocarditis, 8 out of 11 (72.7%) patients received a COVID-19 vaccine; 6 had no documented recurrence of myocarditis post-vaccine. Post-vaccine recurrence was unable to be confirmed in a further two based on the details in the medical record. Three participants had repeat infections after myocarditis with no documented recurrence.

**Conclusion:**

In MHS beneficiaries, the incidence of post-SARS-CoV-2 myocarditis is low and myocarditis complications were infrequent. We did not confirm myocarditis recurring after reinfection or post-myocarditis vaccination in this case series; this finding requires further study.

**Disclosures:**

**Simon Pollett, MBBS**, AstraZeneca: The IDCRP and the Henry M. Jackson Foundation (HJF) were funded to conduct an unrelated phase III COVID-19 monoclonal antibody immunoprophylaxis trial **Mark P. Simons, PhD**, AstraZeneca: The IDCRP and HJF were funded to conduct an unrelated phase III COVID-19 monoclonal antibody immunoprophylaxis trial as part of US Govt COVID Response **David Tribble, MD, DrPH**, AstraZeneca: The IDCRP and HJF were funded to conduct an unrelated phase III COVID-19 monoclonal antibody immunoprophylaxis trial as part of US Govt COVID response **Timothy Burgess, MD, MPH**, AstraZeneca: The IDCRP and the Henry M. Jackson Foundation (HJF) were funded to conduct an unrelated phase III COVID-19 monoclonal antibody immunoprophylaxis trial

